# Integrative 16S rRNA and transcriptome analysis reveals the molecular mechanisms underlying salt- tolerant germination in highland barley (*Hordeum vulgare* var. *coeleste Linnaeus*) seeds

**DOI:** 10.3389/fpls.2025.1691647

**Published:** 2025-11-06

**Authors:** Panrong Ren, Yan Qiao, Jie Wang

**Affiliations:** 1 School of Agriculture and Bioengineering, Longdong University, Qingyang, Gansu, China; 2 College of Life and Environmental Sciences, Hangzhou Normal University, Hangzhou, Zhejiang, China

**Keywords:** highland barley, salt-tolerant germination, transcriptome, 16S rRNA sequencing, RPK gene family

## Abstract

Salt stress is a critical abiotic factor that impairs crop seed germination and limits agricultural productivity. Elucidating the mechanisms governing salt tolerance is essential for development of salt-tolerant crop varieties. In this investigation, 217 accessions of highland barley (*Hordeum vulgare* var. *coeleste Linnaeus*) were evaluated. Germination assays conducted under 200 mmol/L and 500 mmol/L NaCl conditions identified a salt-tolerant variety 37 and a salt-sensitive variety 44. By integrating transcriptome sequencing, 16S rRNA sequencing, and Na^+^/K^+^ content analysis, we systematically investigated the molecular mechanisms underlying salt-tolerant germination in highland barley seeds. Our findings revealed that the salt-tolerant variety 37 maintained a high germination rate of 98% under 500 mmol/L NaCl stress, with lower Na^+^ accumulation (4.24 g/kg) and a lower Na^+^/K^+^ ratio (2.59) compared to the salt-sensitive variety 44 (Na^+^ accumulation: 4.89 g/kg, Na^+^/K^+^ ratio: 3.62). Analysis of 16S rRNA sequencing data showed a significant increase in the abundance of the endophytic bacterium *Brevundimonas* in salt-tolerant variety 37 under high-salt conditions, which was positively correlated with K^+^ content. In contrast, the dominant bacterium *Rhodococcus* in salt-sensitive variety 44 exhibited a positive correlation with Na^+^ content and the Na^+^/K^+^ ratio. Transcriptome sequencing identified 1,467 and 1,644 differentially expressed genes (DEGs) in salt-tolerant variety 37 and salt-sensitive variety 44, respectively. Pathway enrichment analysis indicated that DEGs in salt-tolerant variety 37 were primarily associated with “potassium ion homeostasis” and “response to oxidative stress”. Weighted gene co-expression network analysis (WGCNA) identified 5 co-expression modules, among which the MEyellow module was correlated with Na^+^ content (r = 0.59). Ten core genes were identified, including WRKY transcription factor (*HORVU.MOREX.r3.3HG0268090*) and receptor protein kinase (RPK; *HORVU.MOREX.r3.4HG0331910*). A total of 174 *HvRPK* genes were identified, distributed across 7 chromosomes with a predominant localization on chromosome 2. These genes exhibited functional conservation and were involved in salt stress signaling pathways. Phylogenetic, collinearity, and cis-element analyses further supported their regulatory role in salt stress responses. This study clarifies the key mechanisms underlying salt-tolerant germination in highland barley seeds, providing valuable insights and genetic resources for the molecular breeding of salt-tolerant crops.

## 1 Introduction

Salt stress is a major abiotic factor that restricts global agricultural productivity by adversely affecting crop growth, development, yield, and quality (Zhang et al., 2023). Current data indicate that salinization affects approximately 20% of the world’s arable land and nearly 50% of irrigated areas to varying degrees (Hassani et al., 2021; Zhou et al., 2017). Projections suggest that these figures will escalate by 2050, posing a severe threat to global food security. Highland barley (*Hordeum vulgare* var. *coeleste Linnaeus*), a widely cultivated gramineous crop, serves as a critical resource for food, feed, and the brewing industry (Obadi et al., 2021). Over time, it has evolved specific stress tolerance traits, making it a valuable model for investigating plant salt tolerance mechanisms (Dawson et al., 2015). Among all stages of the plant life cycle, seed germination is particularly vulnerable to salt stress (Wang et al., 2024). Salt stress inhibits seed water uptake, disrupts dormancy, and retards embryo growth through osmotic stress, ion toxicity, and oxidative damage-ultimately leading to reduced germination rates and seedling vigor. Therefore, identifying key genes associated with salt-tolerant germination in highland barley seeds and elucidating their molecular pathways hold significant theoretical and practical importance for developing new salt-tolerant crop varieties and improving the productivity of saline-alkali lands.

The advancement of high-throughput sequencing technology has established transcriptome sequencing as a fundamental tool for deciphering plant stress response mechanisms (Wang et al., 2009; Liu et al., 2021). RNA-seq enables comprehensive exploration of dynamic changes in gene expression under stress conditions and facilitates the identification of key candidate genes. Notably, transcriptome analyses have delineated salt stress-responsive genes and pathways in model plants such as *Arabidopsis thaliana* (Abdelrahman et al., 2021; Wu et al., 2021) and *Oryza sativ* (Cui et al., 2024; Wei et al., 2024). Additionally, 16S rRNA sequencing has provided a novel perspective for investigating plant-microbe interactions (Hrovat et al., 2024). Seed endophytes have been shown to modulate salt tolerance responses by regulating host nutrient uptake, hormone metabolism, and stress signal transduction, thereby underscoring their significant role in enhancing plant stress resistance (Cheng et al., 2021; Wang and Zhang, 2023). Maintaining ion balance, particularly Na^+^ and K^+^ homeostasis, is a pivotal mechanism for plants to cope with salt stress (Ran et al., 2022). Excessive Na^+^ accumulation disrupts cellular ion balance and metabolism, whereas K^+^-as an essential nutrient and signaling molecule-is crucial for maintaining cell osmotic pressure, enzyme activity, and charge balance. Consequently, a multi-omics approach integrating transcriptomics, microbiomics, and ion content analysis offers a powerful strategy to systematically unravel the mechanisms governing salt-tolerant germination in highland barley seeds.

Receptor Protein Kinases (RPKs) are a class of transmembrane proteins localized to the cell membrane, consisting of an extracellular ligand-binding domain, a transmembrane domain, and an intracellular kinase domain (Wei et al., 2020). *RPKs* can perceive external signals and transmit them intracellularly via phosphorylation cascades, thereby regulating various physiological processes including plant growth, immune responses, and stress adaptation (Ding et al., 2024; Liu et al., 2024). Under salt stress, *RPK* family members sense salt signals and activate downstream pathways (e.g., MAPK cascades, ABA signaling) (Shi et al., 2014; Wang et al., 2016), which in turn regulate the expression of salt-tolerance genes, modulate ion transport, promote the synthesis of osmotic adjustment compounds, and scavenge reactive oxygen species (ROS)-ultimately enhancing plant salt tolerance. While numerous *RPK* genes involved in salt stress responses have been characterized in model plants like *Arabidopsis thaliana* (Eckardt, 2009; Kemmerling et al., 2011; Shi et al., 2014) and rice (Cheng et al., 2009; Zhou et al., 2023), comprehensive identification of the *RPK* gene family in highland barley and functional investigations into its role in salt-tolerant seed germination remain limited. Therefore, there is an urgent need to elucidate the underlying mechanisms in this context.

In this study, one salt-tolerant (designated as 37) and one salt-sensitive (designated as 44) were selected from a pool of 217 highland barley accessions. Transcriptome sequencing, 16S rRNA sequencing, and Na^+^/K^+^ content determination were performed on seeds of these two varieties under both control conditions and 500 mmol/L NaCl treatment. We systematically analyzed changes in gene expression and variations in dominant endophytic bacteria in seeds under salt stress, followed by genome-wide screening and analysis of candidate genes (*HvRPK* genes) associated with salt-tolerant germination. The primary objective was to elucidate the mechanisms underlying salt-tolerant germination in highland barley seeds, establish a theoretical framework for investigating its genetic basis for salt tolerance, and offer potential genes and genetic markers for the molecular breeding of novel salt-tolerant varieties.

## 2 Materials and methods

### 2.1 Seed germination experiment

In this study, 217 highland barley accessions collected from diverse regions were selected (**Supplementary Table S1**) and stored in the Biotechnology Laboratory of the School of Agriculture and Bioengineering, Longdong University. Seeds were carefully chosen based on fullness, integrity, and absence of mildew. They were thoroughly cleaned by rinsing under running water for 10 minutes to remove surface contaminants, followed by treatment with 75% ethanol for 40 seconds and subsequent double rinsing with distilled water. Seeds were further disinfected with 5% sodium hypochlorite for 10 minutes and rinsed with distilled water three times. Disinfected seeds were placed on petri dishes lined with two layers of filter paper and treated with NaCl solutions at concentrations of 200 mmol/L and 500 mmol/L. The petri dishes were incubated in a constant-temperature light incubator set at 22°C (16 h light/8 h dark photoperiod; 60% relative humidity). The experimental procedure was replicated three times, with 50 seeds used per replication. Seed germination was defined as radicle emergence, and the number of germinated seeds was recorded daily. Germination rate was calculated on the fifth day using the formula: Germination rate (%) = (Number of germinated seeds on the fifth day/Total number of tested seeds) × 100%. By comparing germination rates under salt stress, variety 37 (salt-tolerant) and variety 44 (salt-sensitive) were selected for subsequent analyses. These two varieties were treated under control conditions (0 mmol/L NaCl) and salt stress (500 mmol/L NaCl) for 3 days. Seed samples were collected for the determination of Na^+^ and K^+^ contents, total DNA extraction (for 16S rRNA amplicon sequencing), and RNA extraction (for transcriptome sequencing).

### 2.2 Determination of sodium and potassium ion contents in highland barley seeds

Dry seed samples (0.10 g) from salt-tolerant variety 37 and salt-sensitive variety 44 were weighed into a polytetrafluoroethylene digestion inner tanks. Five milliliters of nitric acid was added, and the samples were soaked overnight. The tanks were sealed and placed in stainless-steel outer jackets, then subjected to a series of temperature steps: 80°C for 1–2 h, 120°C for 1–2 h, and 160°C for 4 h. After natural cooling, the acid was evaporated until nearly dry. The digested solution was transferred to a 25 mL volumetric flask, and the tank and lid were rinsed thrice with 1% nitric acid solution. The washing were combined with the digested solution, diluted to volume with 1% nitric acid, and thoroughly mixed for subsequent analysis. A reagent blank control was prepared simultaneously. Following sample digestion, an inductively coupled plasma mass spectrometer (ICP-MS) was used for analysis. Qualification was based on the specific mass-to-charge ratio (m/z) of the elements, and quantification was performed using the external standard method (where the ratio of the mass spectrometry signal intensity of the target element to the internal standard signal intensity is directly proportional to the element concentration). The calculation formula was as follows: Content of elements (Na^+^, K^+^) (mg/kg) = C×V/m (C-The concentration of the element measured by the instrument, mg/L; V-Digestion volume, mL; m-The value of the mass of the test sample.).

### 2.3 16S rRNA sequencing

Microbial community DNA was extracted from highland barley seeds using the CTAB method. DNA integrity was assessed via 1% agarose gel electrophoresis, and DNA concentration and purity were determined using a UV spectrophotometer. The V3-V4 variable regions of the 16S rRNA gene were PCR-amplified from the extracted DNA template using primers 341F (5’-CCTACGGGGNGGCWGCAG-3’) and 805R (5’-GACTACHVGGGGTATCTAATCC-3’). The PCR reaction system (25 μL total volume) consisted of: Phusion Hot start flex 2× Master Mix (12.5 μL), each primer (1.25 μL), 50 ng of template DNA, and ddH_2_O (up to 25 μL). The amplification protocol included initial denaturation at 98°C for 30 s, followed by 35 cycles of denaturation at 98°C for 10 s, annealing at 54°C for 30 s, extension at 72°C for 45 s, and a final extension at 72°C for 10 min. The resulting PCR products (~460 bp) were verified by 2% agarose gel electrophoresis, purified using AMPure XT magnetic beads to remove primer dimers and nonspecific products, and analyzed for fragment distribution and integrity using an Agilent 2100 bioanalyzer. Library concentration was determined with an Illumina library quantification kit. Libraries with concentrations >2 nM were selected, diluted to 4 nM, denatured with NaOH, and prepared for sequencing. Paired-end 2×250 bp sequencing was performed on an Illumina NovaSeq 6000 platform to generate raw data. PEAR software was used to assemble the sequences based on overlapping regions, followed by quality filtering (Q30 ≥90%) and chimera removal to obtain clean data. The DADA2 plugin in the QIIME2 platform was utilized for demultiplexing, quality control, denoising, and generation of amplicon sequence variants (ASVs) with single-base precision via dereplication. ASV abundance tables and representative sequences were constructed, and α-diversity analysis was performed. Species classification annotation was conducted using the SILVA database (Release 138) to obtain species composition information from phylum to species levels. Statistical analysis was performed to determine the relative abundance of species in each sample and to identify inter-group differential species.

### 2.4 Transcriptome sequencing and WGCNA analysis

Total RNA was extracted from samples using the TRIzol method (Thermo Fisher, 15596018) and subsequently purified. RNA quality was assessed using an Agilent 2100 Bioanalyzer and the RNA 6000 Nano LabChip Kit (Agilent, 5067-1511), with a minimum RNA integrity number (RIN) threshold of >7.0 required for library construction. To enrich mRNA, five micrograms of total RNA was subjected to two rounds of purification using Dynabeads Oligo (dT) (Thermo Fisher, cat. 25-61005). The purified mRNA was fragmented by incubation with the Magnesium RNA Fragmentation Module (NEB, e6150) at 94°C for 5–7 minutes. Subsequently, first-strand cDNA was synthesized using SuperScript™ II Reverse Transcriptase (Invitrogen, 1896649) with the fragmented mRNA as a template. U-labeled double-stranded cDNA was synthesized using *E. coli* DNA polymerase I (NEB, m0209), RNase H (NEB, m0297), and dUTP Solution (Thermo Fisher, R0133). The double-stranded cDNA was processed for end repair, A-tail addition, and ligation with dual-index adapters featuring T-base overhangs, followed by size selection using AMPureXP beads. The U-labeled second-strand cDNA was treated with UDG enzyme (NEB, m0280). A cDNA library with fragment sizes of 300 bp ± 50 bp was generated through PCR amplification: initial denaturation at 95°C for 3 minutes; 8 cycles of denaturation at 98°C for 15 seconds, annealing at 60°C for 15 seconds, extension at 72°C for 30 seconds; final extension at 72°C for 5 minutes. Paired-end sequencing (PE150) with 2×150 bp was performed on the Illumina Novaseq™ 6000 platform.

Cutadapt (version 1.9) was utilized to filter reads containing adapters, polyA/polyG, with over 5% unknown nucleotides (N), or over 20% low-quality bases (Q ≤ 20). FastQC (version 0.11.9) was employed to assess sequence quality, and Trimmomatic was used to eliminate adapter sequences. HISAT2 (version 2.2.1) was employed to align clean reads to the reference genome available at https://ftp.ebi.ac.uk/ensemblgenomes/pub/release-61/plants/fasta/hordeum_vulgare/dna/, allowing a maximum of 20 alignments and 2 mismatches per read. StringTie (version 2.1.6) was utilized to assemble the mapped reads of each sample, and gffcompare (version 0.9.8) was used to merge the transcriptomes of all samples. Subsequently, StringTie (version 2.1.6) and ballgown (version 3.4.0) were used to compute the FPKM values of mRNAs for expression level quantification. Differential expression analysis was conducted using DESeq2 (version 3.11), with the criteria for differentially expressed genes (DEGs) set at |log_2_(fold change)| ≥ 4 and adjusted *P*-value < 0.01. Functional enrichment analysis of DEGs was carried out using the AgriGO2 online platform (http://systemsbiology.cau.edu.cn/agriGOv2) to elucidate the biological processes in which they participate. WGCNA analysis was performed using Tbtools software (version 2.034). The DEG expression matrix was input into the WGCNA plugin, followed by the removal of outlier samples via cluster analysis. The soft threshold was selected to establish a scale-free network, based on the minimum power value with an R^2^ correlation coefficient > 0.8 or at the plateau stage. Modules were generated with default settings (minimum module size: 30, trimming height: 0.5), resulting in the classification of DEGs into 5 modules. Subsequently, correlation analysis was performed between each module and Na^+^ content. Modules significantly linked to the target trait were identified by assessing the module eigengene (ME) values. Core genes were identified by considering the significance and connectivity of genes within the modules. The regulatory network was visualized using Cytoscape (version 3.10.1).

### 2.5 Identification of the *RPK* gene family in barley

The genome sequence of barley was retrieved from the EnsemblPlants database (https://ftp.ensemblgenomes.ebi.ac.uk/pub/plants/release-61/fasta/hordeum_vulgare/dna/, accessed on July 20, 2025), while protein sequences of RPK family members from wheat (*T. aestivum*), rice (*O. sativa*), maize (*Z. mays*), sorghum (*S. bicolor*), *Brachypodium distachyon*, and *Arabidopsis thaliana* were obtained from the NCBI database (http://www.ncbi.nlm.nih.gov/, accessed on July 20, 2025). Alignment of these RPK protein sequences with the genome database using the tblastn method identified *RPK* genes in highland barley. The gene accession numbers, coding sequence lengths, and the number of amino acids were obtained from the genome database; the molecular weight and isoelectric point were calculated through Expasy (https://www.expasy.org/resources/protparam, accessed on July 20, 2025); transmembrane helices were predicted using TMHMM - 2.0 (https://services.healthtech.dtu.dk/services/TMHMM-2.0/, accessed on July 20, 2025); signal peptides were predicted using SignalP - 4.1 (https://services.healthtech.dtu.dk/services/SignalP-4.1/, accessed on July 20, 2025).

Amino acid sequences of HvRPK protein were aligned using the ClustalW method in MEGA 7 software. A phylogenetic tree was constructed using the neighbor-joining (NJ) method and visualized on the iTOL website (https://itol.embl.de/upload.cgi, accessed on July 22, 2025). To assess collinearity and chromosomal localization of *HvRPK* family genes, annotation information and whole-genome protein sequences of wheat, rice, maize, sorghum, *Brachypodium distachyon*, and *Arabidopsis thaliana* were obtained from the EnsemblPlants database (https://plants.ensembl.org/index.html, accessed on July 23, 2024). The collinearity relationships of *RPK* genes between highland barley and other species were determined and visualized using the MCScanX toolkit in TBtools with default parameters.

The whole-genome sequence and CDS sequence of the *HvRPK* gene were downloaded to analyze gene structure, domains, motifs, and cis-regulatory elements. Gene structure analysis was performed using TBtools. Protein motifs were identified using the MEME website (http://meme-suite.org/tools/meme, accessed on July 24, 2025), and protein domains were analyzed using Batch CD-Search (https://www.ncbi.nlm.nih.gov/Structure/bwrpsb/bwrpsb.cgi, accessed on July 24, 2025). The 2000-bp promoter sequence upstream of the ATG start codon was extracted and analyzed for cis-elements using Plant CARE (http://bioinformatics.psb.ugent.be/webtools/plantcare/html/, accessed on July 26, 2025).

### 2.6 Real-time quantitative PCR

To assess the response of the 10 screened *HvRPK* genes to high-salt stress during highland barley seed germination, seeds treated with high-salt stress for 3 days were collected (1 g), immediately frozen in liquid nitrogen, and ground into powder. RNA was isolated using TRIzol reagent (Invitrogen, 15596026), followed by cDNA synthesis from 1.5 µg of total RNA using the PrimeScript^®^ RT reagent Kit (TaKaRa, Dalian). The diluted cDNA was used as a template for qRT-PCR analysis with three biological replicates. The thermal cycling conditions comprised an initial denaturation step at 95°C for 30 s, followed by 40 cycles of denaturation at 95°C for 5 s and annealing/extension at 58°C for 30 s. Subsequently, a melting curve analysis was conducted (95°C for 15 s, 58°C for 30 s, 95°C for 15 s). *HvActin* served as the internal control gene, and the relative gene expression was determined using the 2^-ΔΔCt^ method. The qRT-PCR primers were designed through NCBI Primer-BLAST (**Supplementary Table S2**).

## 3 Results

### 3.1 Screening of salt-tolerant variety 37 and salt-sensitive variety 44

**Figure 1:**
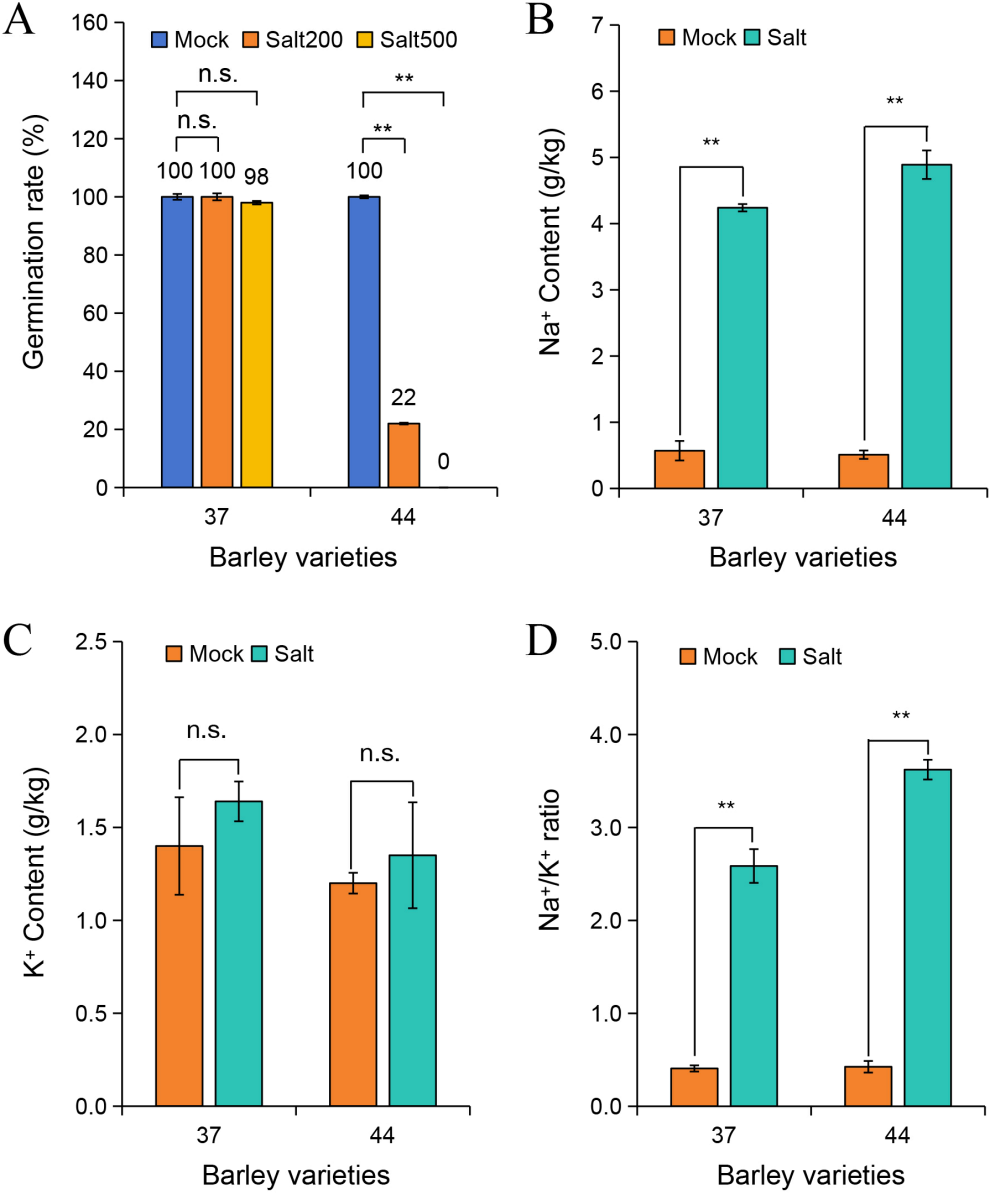
Effects of salt stress on germination rate and ion content in different barley varieties. **(A)** Germination rates of barley varieties 37 and 44 under 0 mmol/L (Mock), 200 mmol/L (Salt200), and 500 mmol/L (Salt500) NaCl treatments. Na^+^ content **(B)**, K^+^ content **(C)**, and Na^+^/K^+^ ratio **(D)** in barley varieties 37 and 44 under Mock (0 mmol/L NaCl) and Salt (500 mmol/L NaCl) treatments. Differences among treatments within each variety were evaluated with paired t-tests. The smaller bars are standard error of means (n=3). **indicates a significant difference at *P* < 0.01; n.s. indicates no significant difference.

To elucidate the salt tolerance characteristics of different highland barley accessions, 217 samples from diverse regions were exposed to 200 mmol/L and 500 mmol/L NaCl stress. The germination progress was monitored over 5 days, and germination rates were calculated (**Supplementary Table S1**). The findings depicted in **Figure 1A** revealed that under normal conditions, both variety 37 and variety 44 exhibited a 100% germination rate. When subjected to 200 mmol/L NaCl stress, variety 37 maintained a 100% germination rate, whereas variety 44 showed a significant decline to 22%. Under 500 mmol/L NaCl stress, variety 37 retained a germination rate of 98%, while variety 44 failed to germinate (**Supplementary Figure S1**). Thus, variety 37 (salt-tolerant) and variety 44 (salt-sensitive) were selected for subsequent analyses.

### 3.2 Comparison of sodium and potassium content in barley seeds

Na^+^ contents in salt-tolerant variety 37 and salt-sensitive variety 44 were quantified after 3 days of treatment under control (0 mmol/L NaCl) and 500 mmol/L NaCl conditions (**Figure 1B**). Under salt stress, Na^+^ content significantly increased in seeds of both varieties compared to the control group (*P* < 0.01). Specifically, Na^+^ content in salt-tolerant variety 37 was 4.24 g/kg, which was lower than the 4.89 g/kg observed in salt-sensitive variety 44. Analysis of K^+^ content showed no significant difference between salt-stressed and control groups in either variety (**Figure 1C**). Assessment of the Na^+^/K^+^ ratio demonstrated a significant increase under salt stress in both varieties (*P* < 0.01), with the ratio in salt-sensitive variety 44 (3.62) being higher than that in salt-tolerant variety 37 (2.59) (**Figure 1D**). This discrepancy suggests a more severe ion imbalance in the salt-sensitive variety under high-salt conditions.

### 3.3 Analysis of seed endophytic microbial communities in highland barley varieties under salt stress

To investigate the impact of seed endophytes on the salt tolerance of highland barley, 16S rRNA amplicon sequencing was performed on the salt-tolerant variety 37 and salt-sensitive variety 44. The raw sequencing data were subjected to paired-end merging, quality assessment, and chimera removal, followed by statistical analysis of the refined dataset (**Supplementary Table S3**). The Raw_Tags counts exhibited minimal variation across samples, ranging from 80,159 to 87,554. After quality control and chimera removal, the Valid_Tags counts ranged from 72,363 to 80,077, with the lowest Valid% (Valid_Tags/Raw_Tags) being 89.96%-indicating that most high-quality data were retained through filtering. The lowest Q20 and Q30 values across all samples were 97.09% and 91.58%, respectively, affirming the accuracy and reliability of the sequencing data. Furthermore, an Alpha diversity analysis of each sample (**Supplementary Table S4**) revealed significant variations in the number of observed_otus. Sample 44Mock2 exhibited the fewest observed_otus, with only 28, while sample 37Mock1 displayed the highest count at 376. The consistency of the Chao1 index with the observed_otus count in most samples suggests adequate sequencing depth, likely encompassing the species diversity effectively. A higher Shannon value signifies greater species diversity and a more uniform distribution. The Shannon values ranged from 1.16 to 4.93 across all samples. Sample 37Mock1 demonstrated a relatively high Shannon value, indicating rich and evenly distributed species, whereas sample 44Salt3 showed a low Shannon value, potentially indicating a concentrated species distribution. The analyses of the Simpson and Pielou_e indices aligned with the Shannon index results. The Goods_coverage index, assessing sequencing depth, indicated values at 1 for all samples, signifying sufficient depth to accurately depict microbial community structures.

**Figure 2:**
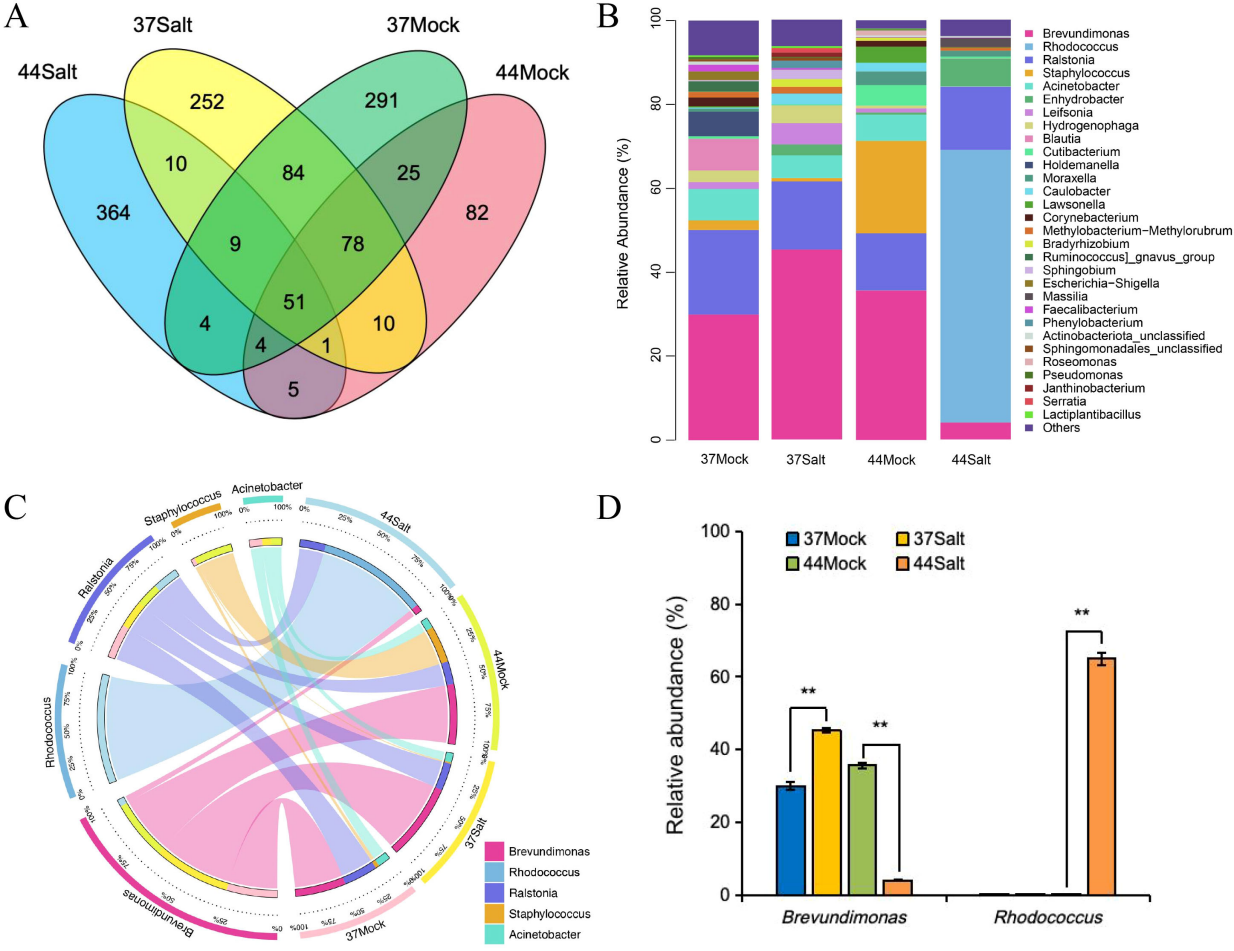
Analysis of microbial communities in different barley varieties (37 and 44) under mock (0 mmol/L NaCl) and salt stress (500 mmol/L NaCl). **(A)** Venn diagram showing shared and unique ASVs. **(B)** Relative abundance of microbial genera. **(C)** Distribution and correlation of five key microbial genera. **(D)** Relative abundance of *Brevundimonas* and *Rhodococcus*. Differences among treatments within each variety were evaluated with paired t-tests. The smaller bars are standard error of means (n=3). **indicates a significant difference at *P* < 0.01.

Venn diagram analysis of ASVs at the genus level revealed 51 shared ASVs across all samples, suggesting their involvement in fundamental ecosystem functions such as material cycling and stress adaptability. After salt treatment, the salt-tolerant variety 37 and the salt-sensitive variety 44 exhibited 252 and 364 unique ASVs (**Figure 2A**), respectively, indicating the enrichment of specific microbial taxa in response to salt stress. Analysis of microbial community composition demonstrated that *Brevundimonas* was the dominant genus in the salt-tolerant variety 37, while *Rhodococcus* prevailed in the salt-sensitive variety 44. Notably, salt stress led to the disappearance of certain rare genera and a decrease in community diversity in the salt-tolerant variety 37, whereas low-abundance genera were eliminated in the salt-sensitive variety 44, resulting in a more uniform community structure (**Figure 2B**; **Supplementary Figures S2**, **S3**). The abundance of salt-tolerance-related genera increased under salt stress in the salt-tolerant barley but decreased in the salt-sensitive barley, indicating differences in salt tolerance adaptability. Analysis of the five dominant genera (**Figure 2C**) revealed that *Brevundimonas* and *Rhodococcus* were associated with the salt-tolerant and salt-sensitive barley varieties, respectively, with their abundances increasing under salt stress. *Ralstonia* exhibited no significant changes under control and salt stress conditions in both barley varieties. *Staphylococcus* was enriched only in the control group of the salt-sensitive variety, while *Acinetobacter* disappeared after salt stress in the salt-sensitive barley. Correlation analysis showed a positive association between *Rhodococcus* and Na^+^ content and Na^+^/K^+^ ratio, and a positive correlation between *Brevundimona*s and K^+^ content (**Supplementary Figure S4**). The abundance of *Brevundimonas* increased from 30.00% to 45.27% in the salt-tolerant barley under salt stress, whereas it decreased from 35.63% to 4.05% in the salt-sensitive barley. *Rhodococcus* abundance significantly rose to 64.96% only in the salt-sensitive barley under salt stress, with no substantial change in the salt-tolerant barley (**Figure 2D**).

### 3.4 Transcriptome sequencing to identify salt-tolerant candidate genes

**Figure 3:**
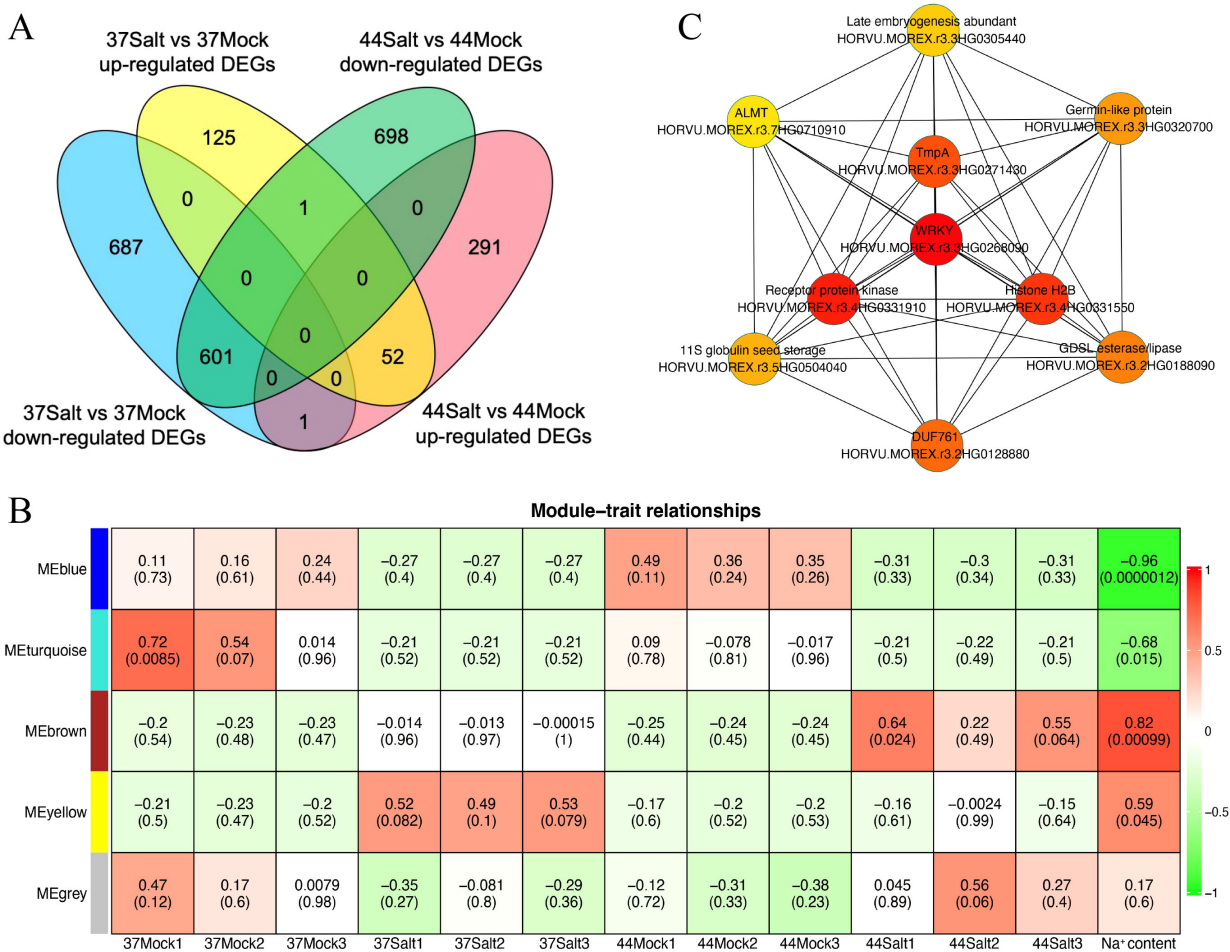
Analysis of DEGs and gene co-expression modules in barley under salt stress. **(A)** Venn diagram of up-regulated and down-regulated DEGs between 37Salt vs 37Mock and 44Salt vs 44Mock comparisons. **(B)** Heatmap of module-trait relationships (The color ranging from red to green indicates that the correlation coefficient changes from 1 to -1). **(C)** Ten core genes identified in the MEyellow module (the node color ranging from red to yellow indicates that the gene connectivity within the module decreases from high to low).

Transcriptome sequencing data were used to identify DEGs in highland barley varieties under control and salt stress conditions, using strict criteria of |log_2_(fold change)| > 4 and qvalue < 0.01. After salt treatment, salt-tolerant variety 37 had 178 up-regulated and 1,289 down-regulated genes compared to the control, while salt-sensitive variety 44 had 344 up-regulated and 130 down-regulated genes (**Figure 3A**). In salt-tolerant variety 37, the DEGs were predominantly associated with GO pathways related to ion balance, oxidative stress, and responses to diverse environmental stresses, encompassing terms such as “potassium ion homeostasis (GO:0055075)”, “response to oxidative stress (GO:0006979)”, “response to stress (GO:0006950)”, “defense response (GO:0006952)”, and “response to stimulus (GO:0050896)” (**Supplementary Figure S5**). Conversely, in salt-sensitive highland barley variety 44, the DEGs were primarily enriched in GO pathways linked to stimulus response and substance metabolism, including terms like “response to stimulus (GO:0050896)”, “response to stress (GO:0006950)”, “sucrose catabolic process (GO:0005987)”, “starch catabolic process (GO:0005983)”, “lignin catabolic process (GO:0046274)”, and “cell wall biogenesis (GO:0042546)” (**Supplementary Figure S6**).

The WGCNA method was used to identify salt-tolerant candidate genes by correlating DEGs with Na^+^ content. Initially, a phylogenetic tree was constructed using the expression matrix of DEGs across all samples, with the removal of any identified abnormal samples, of which none were detected in this study. Subsequently, the soft threshold was set at 20 based on scale independence and mean connectivity analyses. The scale-free network’s goodness-of-fit (scale free topology model fit, signed R^2^) approached 0.8, indicating stable mean connectivity (**Supplementary Figure S7**). The network constructed with a soft threshold of 20 displayed scale-free characteristics in the soft connectivity distribution. The scale-free topology test revealed a linear fitting R^2^ of 0.87 and a slope of -0.86, confirming the network’s adherence to scale-free topology and suitability for module identification (**Supplementary Figure S8**). Genes exhibiting similar expression patterns were clustered into 5 modules based on co-expression patterns (**Supplementary Figure S9**). Correlation analysis between each module and Na^+^ content identified the MEbrown and MEyellow modules as significantly positively correlated with the target trait, with correlation coefficients of 0.82 and 0.59, respectively (**Figure 3B**). Additionally, correlation analysis between modules and germination rate showed no significant relationship between MEyellow module and germination rate (correlation coefficient = 0.18, *P* = 0.57; **Supplementary Figure S10**). Further analysis of module gene expression in salt-tolerant variety 37 under salt stress highlighted the key role of the MEyellow module in the salt-tolerant germination process of highland barley seeds. Subsequently, the top 10 core genes within the MEyellow module were identified based on gene connectivity, including *HORVU.MOREX.r3.3HG0268090* (WRKY transcription factor), *HORVU.MOREX.r3.4HG0331910* (Receptor protein kinase), *HORVU.MOREX.r3.4HG0331550* (Histone H2B), *HORVU.MOREX.r3.3HG0271430* (Integral membrane protein TmpA), *HORVU.MOREX.r3.2HG0128880* (cotton fiber, putative DUF761), *HORVU.MOREX.r3.2HG0188090* (GDSL esterase/lipase), *HORVU.MOREX.r3.3HG0320700* (Germin-like protein 1-1), *HORVU.MOREX.r3.5HG0504040* (11S globulin seed storage protein 2), *HORVU.MOREX.r3.3HG0305440* (Late embryogenesis abundant protein), and *HORVU.MOREX.r3.7HG0710910* (Aluminum-activated malate transporter-like) (**Figure 3C**).

### 3.5 Genome-wide identification of the RPK gene family in highland barley

In order to validate the involvement of candidate genes in salt stress response, the *RPK* gene family was subjected to comprehensive analysis. A total of 174 *RPK* family genes were identified within the highland barley genome and designated *HvRPK1* to *HvRPK174*. These genes are distributed across 7 chromosomes (1H-7H) of highland barley, with varying copy numbers: 16 genes on chromosome 1H, 40 on chromosome 2H, 13 on chromosome 3H, 19 on chromosome 4H, 20 on chromosome 5H, 28 on chromosome 6H, and 38 on chromosome 7H. Notably, chromosome 2H exhibits the highest density of *RPK* genes, while chromosome 3H displays relatively fewer gene occurrences (**Supplementary Figure S11**). Although the majority of genes are dispersed across chromosomes, instances of gene clustering are evident on specific chromosomes such as 2H and 6H, suggesting potential gene duplication events in these genomic regions.

Analysis of the physicochemical properties of 174 HvRPK proteins (**Supplementary Table S5**) revealed variations in amino acid lengths, ranging from 728 to 1,299 aa. HvRPK68 exhibited the highest amino acid count (1,299 aa), while HvRPK98 had the lowest (728 aa). Molecular weights ranged from 80.39 to 142.07 kDa. Isoelectric points (pI) ranged from 5.32 to 8.68, with 40 genes having a pI < 6.0 (slightly acidic) and 134 genes with a pI ≥ 6.0 (slightly neutral or alkaline). The instability index ranged from 26.69 to 50.85: HvRPK123 had the highest instability index (50.85) and HvRPK78 had the lowest (26.69). Notably, 125 proteins exhibited an instability index < 40, indicative of stability or relatively high stability. The aliphatic index ranged from 78.21 to 118.22, averaging 101.07, suggesting elevated thermal stability among most HvRPK proteins. GRAVY values ranged from -0.237 to 0.21, with 74 proteins having GRAVY values < 0 (hydrophilic) and 100 proteins with GRAVY values ≥ 0 (hydrophobic or amphipathic). TMHMM analysis revealed variability in the number of transmembrane helices among the 174 HvRPK proteins, with 114 proteins possessing one, 46 proteins having two, 6 proteins containing three or more, and 8 proteins lacking transmembrane helices. The presence of transmembrane helices suggests potential localization to the cell membrane and involvement in signal transduction. SignalP prediction identified N-terminal signal peptides in 140 HvRPK proteins, with S-scores ranging from 0.433 to 0.981 and C-scores from 0.169 to 0.889, indicating potential targeting to the cell membrane for functionality. Additionally, 34 proteins lacked signal peptides, suggesting alternative localization mechanisms.

**Figure 4:**
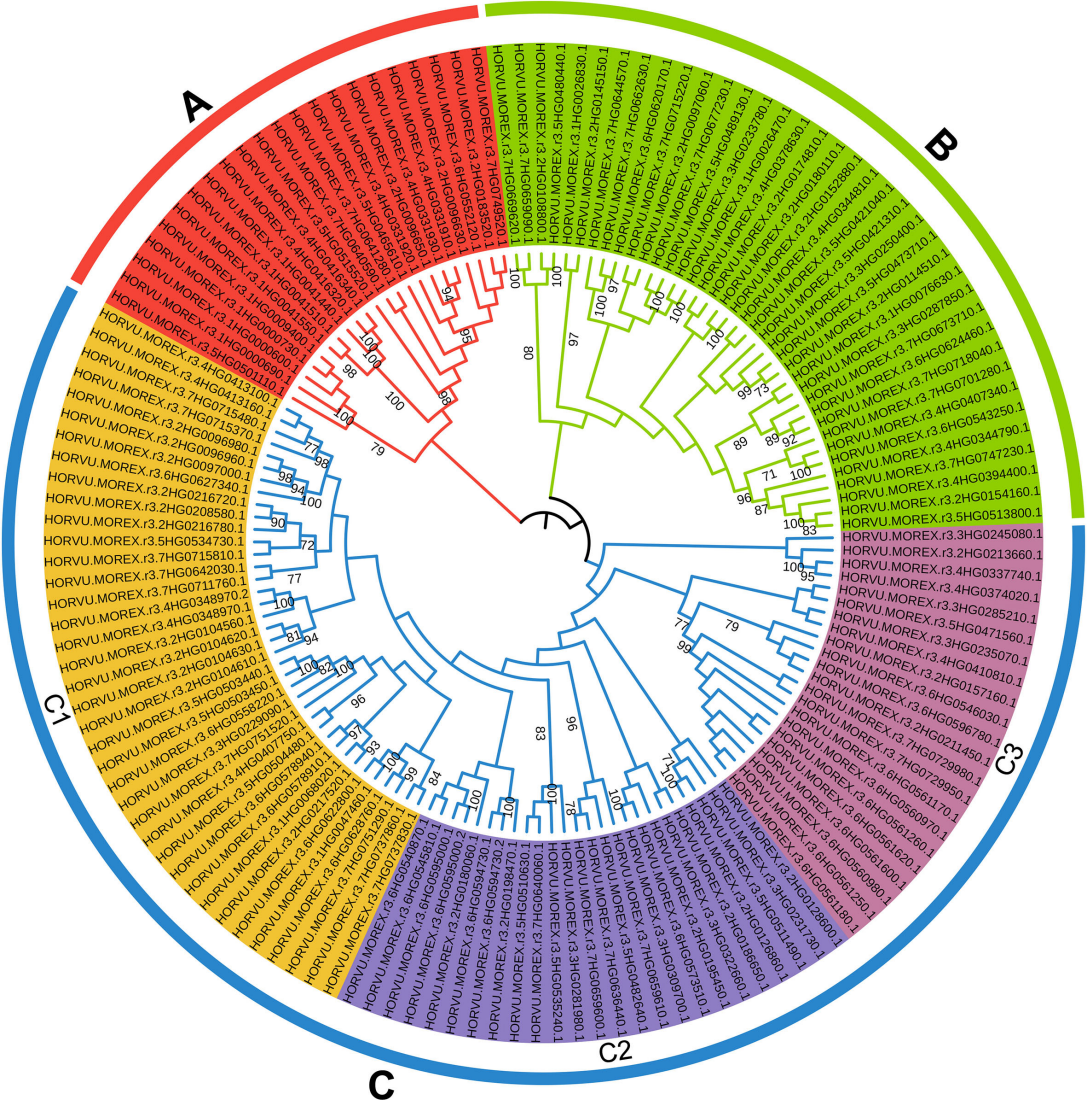
Phylogenetic analysis and classification of *HvRPK* genes. Different colored regions [**(A)** red, **(B)** green, **(C)**] blue, C1-yellow, C2-lavender blue, C3-magenta) represent distinct gene clusters.

In addition, 174 *HvRPK* genes in highland barley were analyzed for evolutionary relationships and classification characteristics. Incomplete genes were filtered out based on amino acid sequence motif analysis, resulting in the retention of 145 *HvRPK* genes. A phylogenetic tree was constructed using the Neighbor-Joining (NJ) method, revealing three distinct groups (A, B, and C) among the 145 *HvRPK* genes (**Figure 4**). Group A comprised 22 *HvRPK* genes exhibiting close clustering in the phylogenetic tree with high bootstrap values, indicating significant similarity in amino acid sequence and structure among the encoded proteins. Group B consisted of 38 *HvRPK* genes, including members like *HORVU.MOREX.r3.2HG0123450* and *HORVU.MOREX.r3.2HG0135670*, potentially involved in the coordinated response of highland barley to biotic or abiotic stresses. Group C encompassed 85 *HvRPK* genes, further categorized into three subgroups: C1 (38 genes), C2 (25 genes), and C3 (22 genes). Genes within each subgroup shared closer evolutionary origins and functional characteristics, potentially contributing to specific physiological processes in highland barley, such as cell proliferation and cell wall synthesis.

**Figure 5:**
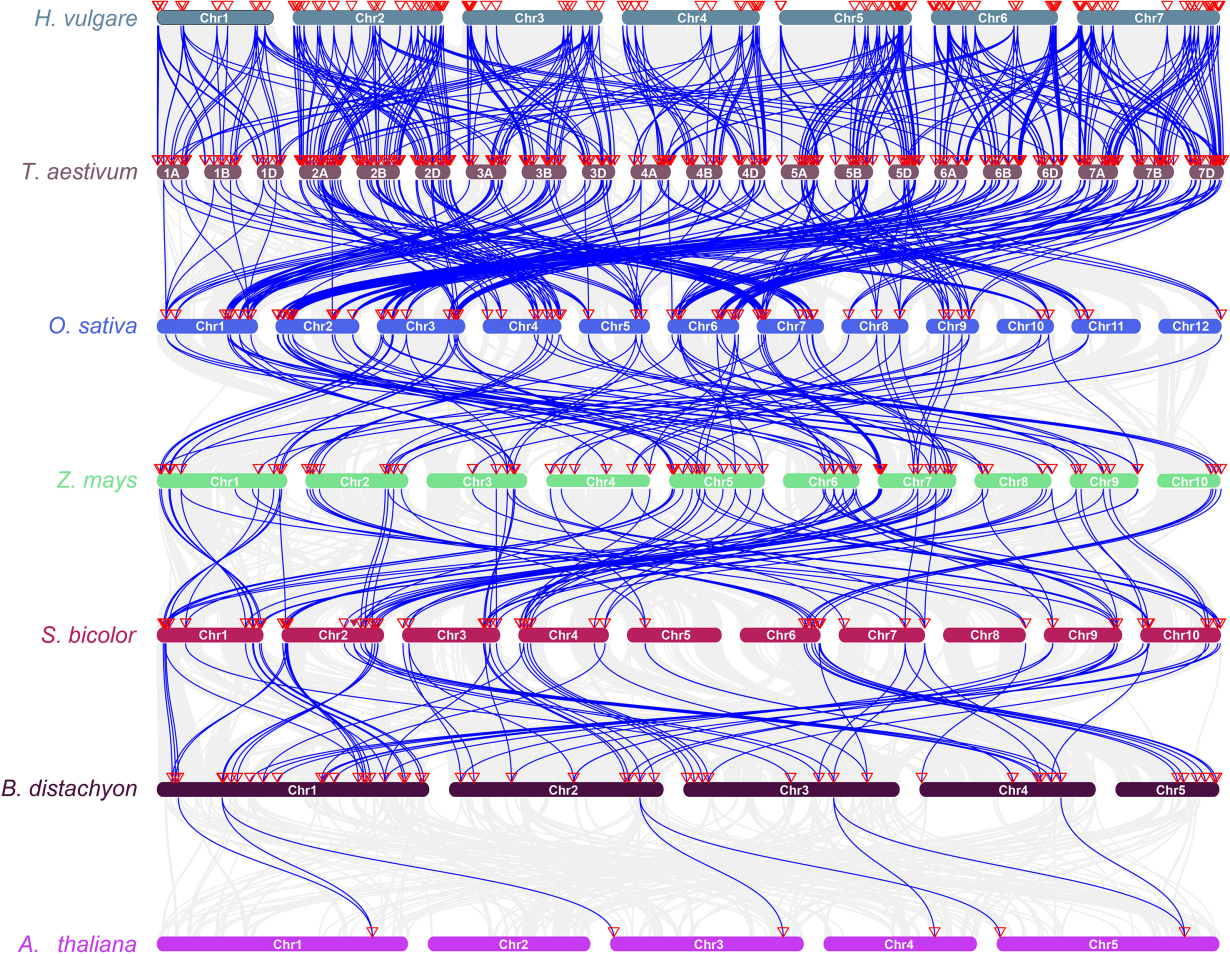
Synteny analysis of *RPK* genes among different plant species. Blue lines represent syntenic relationships between chromosomal regions of different species, and red triangles on chromosomes indicate the positions of *RPK* genes. *Hordeum vulgare* (*H. vulgare*)*, Triticum aestivum* (*T. aestivum*)*, Oryza sativa* (*O. sativa*)*, Zea mays* (*Z. mays*)*, Sorghum bicolor* (*S. bicolor*)*, Brachypodium distachyon* (*B. distachyon*)*, Arabidopsis thaliana* (*A. thaliana*).

To investigate the conservation and evolutionary trends of the *RPK* gene family across species, collinearity analysis was performed on *RPK* genes in highland barley (*H. vulgare* var. *coeleste Linnaeus*), wheat (*T. aestivum*), rice (*O. sativa*), maize (*Z. mays*), sorghum (*S. bicolo*r), *Brachypodium distachyon*, and *Arabidopsis thaliana* (**Figure 5**). The chromosomal collinearity map revealed a strong collinearity relationship between highland barley and other gramineous crops like wheat, rice, and maize. This observation suggests that throughout the evolution of gramineous plants, there is a relatively high level of genomic position conservation and sequence similarity among *RPK* genes, potentially stemming from the inheritance and retention of genes from a common ancestor. Additionally, a subset of collinear genes was shared between highland barley, wheat, and other species, including *Arabidopsis thaliana* (a model dicotyledonous plant). This finding implies that some *RPK* genes originated in the early stages of angiosperm evolution and exhibit functional conservation across species.

Comprehensive analysis of the structural characteristics and functions of 174 *HvRPK* genes was performed by examining their exon-intron structures. The number of exons varied from 1 to 27. Specifically, 52 *HvRPK* genes exhibited a simple structure with only 1 exon, while *HvRPK146* displayed a complex gene structure with 27 exons. The number of introns also varied significantly, ranging from 0 to 26 (**Supplementary Figure S12**), indicating complex gene rearrangement and mutation events during the evolution of *RPK* genes, potentially linked to their functional diversification. Additionally, the conserved domains of HvRPK proteins were identified using the Pfam and SMART databases. All 174 HvRPK proteins contained a protein kinase domain-essential for kinase activity and involvement in phosphorylation signal transduction. Moreover, approximately 83% of the proteins contained a Leucine-Rich Repeat (LRR) domain, which mediates protein-protein interactions and recognizes external signal molecules to activate receptors. Some proteins also harbored additional domains such as the D-mannose binding lectin domain, S-locus glycoprotein domain, and Plant PAN/APPLE-like domain, enhancing the functional diversity of RPK proteins (**Supplementary Figure S13**). Furthermore, analysis using MEME software identified 20 conserved motifs (designated Motif 1 to Motif 20) among HvRPK proteins, with motif lengths ranging from 11 to 50 amino acids. Motif 1 to Motif 13, Motif 18, and Motif 19 were present in most HvRPK proteins, with an occurrence frequency exceeding 81%, likely associated with fundamental functions like kinase activity and substrate binding. In contrast, Motif 14, Motif 15, Motif 16, Motif 17, and Motif 20 exhibited differential distributions, with some motifs specific to certain HvRPK protein sub-families or clusters (**Supplementary Figure S14**).

Cis-acting elements in the promoter regions (2000 bp upstream of the transcription start site) of 174 *HvRPK* genes were predicted and analyzed. Fifteen distinct types of elements associated with growth, development, hormone response, and stress adaptation were identified (**Supplementary Figure S15**). 1) Hormone-responsive elements: Auxin-responsive elements (present in 76 genes, 43.68%): including auxin-responsive elements and cis-acting regulatory elements involved in auxin responsiveness. This suggests potential regulation of these genes by auxin, implicating their involvement in plant growth and stress responses; Abscisic acid (ABA)-responsive elements (present in 148 genes, 85.06%): Given ABA’s pivotal role in stress signaling, the prevalence of its responsive elements suggests the potential involvement of *HvRPK* genes in stress signal transduction, such as drought and salt stress; Methyl jasmonate (MeJA)-responsive elements (present in 140 genes, 80.46%): indicating potential participation of most *HvRPK* genes in defense responses and stress adaptation through the MeJA pathway; Gibberellin-responsive elements (present in 84 genes, 48.28%): potentially contributing to the balance between gibberellin-mediated growth regulation and stress tolerance. 2) Stress and defense-responsive elements: Defense and stress-responsive elements (present in 39 genes, 22.41%): directly linked to the plant’s fundamental defense against biotic and abiotic stresses; Drought-responsive elements (present in 81 genes, 46.55%): specifically the MYB binding site involved in drought-inducibility, suggesting a potential regulatory role of *HvRPK* genes in drought stress response through MYB transcription factors. Low-temperature-responsive elements (present in 71 genes, 40.80%): indicating potential involvement of some *HvRPK* genes in adapting to low-temperature stress. 3) Light-responsive and growth and development elements: Light-responsive elements (present in 124 genes, 71.26%): encompassing cis-acting elements involved in light responsiveness and light-responsive elements, suggesting potential regulation of *HvRPK* gene expression by light signals, implicating their participation in light-dependent growth and development processes; Seed-specific regulatory elements (present in 27 genes, 15.52%): potentially associated with *HvRPK* gene expression during seed development or germination; Meristem expression elements (present in 83 genes, 47.70%): hinting at potential role in plant meristem growth.

### 3.6 Expression of 10 core genes under salt stress

**Figure 6:**
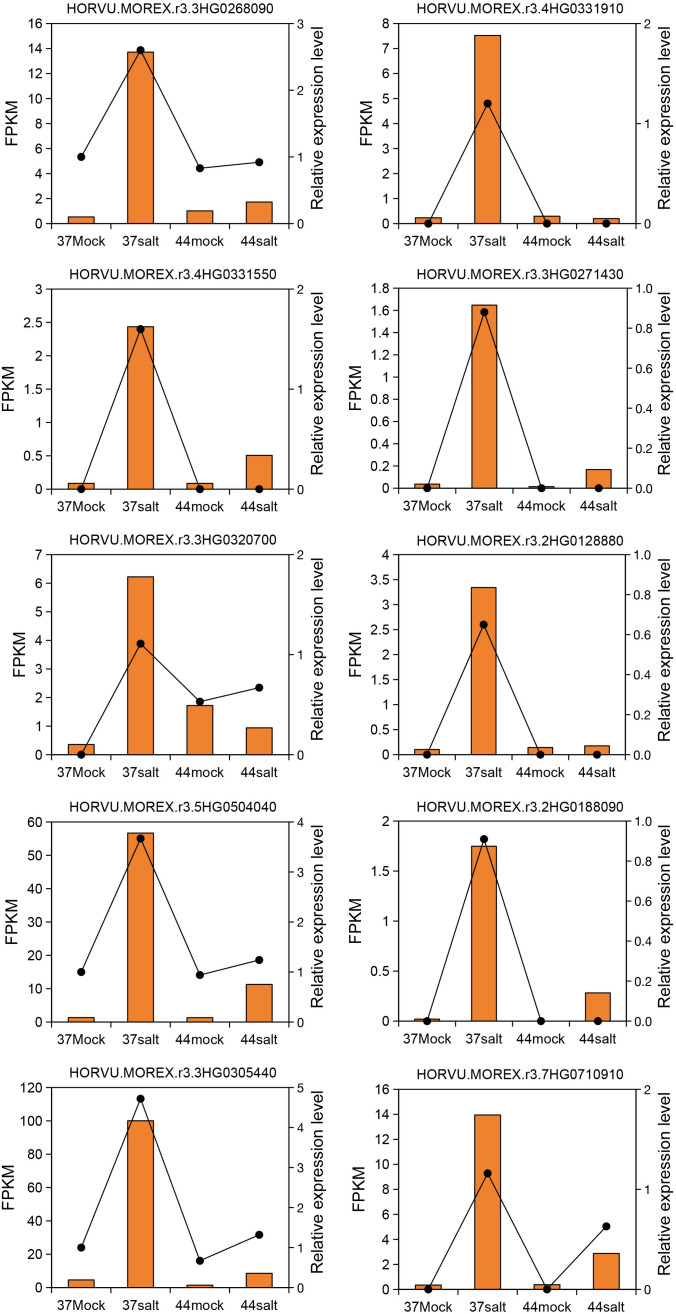
Quantitative reverse-transcription PCR validation of 10 core genes. Yellow columns represent FPKM values from Illumina sequencing. Black dashed lines represent relative expression levels determined by qRT-PCR.

Expression changes of 10 core genes (*HORVU.MOREX.r3.4HG0331910*, *HORVU.MOREX.r3.3HG0268090*, *HORVU.MOREX.r3.4HG0331550*, *HORVU.MOREX.r3.3HG0271430*, *HORVU.MOREX.r3.2HG0128880*, *HORVU.MOREX.r3.3HG0320700*, *HORVU.MOREX.r3.2HG0188090*, *HORVU.MOREX.r3.5HG0504040*, *HORVU.MOREX.r3.3HG0305440*, *HORVU.MOREX.r3.7HG0710910*) in the salt-tolerant variety 37 and the salt-sensitive variety 44 were evaluated using qRT-PCR (relative expression level) and RNA-seq (FPKM value). The results demonstrated a high consistency in expression patterns between qRT-PCR and RNA-seq data. These genes were up-regulated under salt stress in the salt-tolerant variety 37, whereas minimal differences were observed between control and salt stress conditions in the salt-sensitive variety 44 (**Figure 6**).

## 4 Discussion

In this study, a comprehensive approach integrating 16S rRNA sequencing and transcriptome analysis with physiological index determination was used to investigate the molecular mechanisms underlying salt-tolerant germination in highland barley seeds. Two varieties, salt-tolerant 37 and salt-sensitive 44, were identified. Differences in salt tolerance between these varieties were elucidated by analyzing ion balance, endophytic bacterial community composition, and gene expression regulation. These findings provide novel insights into the genetic basis of salt tolerance in highland barley.

### 4.1 Association between salt tolerance phenotype and ion balance in highland barley seeds

Sensitivity of seed germination to salt stress is a crucial determinant of plant salt tolerance (Munns and Tester, 2008; Shu et al., 2017). In this investigation, the salt-tolerant variety 37 maintained a high germination rate of 98% even under 500 mmol/L NaCl stress, whereas the salt-sensitive variety 44 failed to germinate. Khan et al. revealed that the salt-tolerant pea varieties (BD4175 and BD4225) exhibited superior performance under 8 dS/m salt stress, whereas the salt-sensitive varieties (BD4182 and BD6944) displayed the lowest germination rates (Khan et al., 2022). Li et al. demonstrated that the germination rates of both the salt-tolerant variety FL478 and salt-sensitive variety IR29 rice varieties were inhibited under 150 mmol/L NaCl stress, but the reduction was less severe in FL478 (Li and Guo, 2023). Maintaining Na^+^ and K^+^ balance is a key mechanism for plants to withstand salt stress. Our findings revealed a notable increase in Na^+^ content in the seeds of both varieties under high salinity conditions. However, the salt-sensitive variety 44 exhibited higher Na^+^ accumulation (4.89 g/kg) compared to the salt-tolerant variety 37 (4.24 g/kg), resulting in a significantly higher Na^+^/K^+^ ratio of 3.62 in 44, in contrast to 2.59 in 37. This indicates a more severe ion imbalance in the salt-sensitive variety 44. This observation is consistent with previous studies suggesting that “salt-tolerant plants alleviate ion imbalance by limiting Na^+^ accumulation or maintaining high K^+^ levels” (Amorim et al., 2023; Feng et al., 2025). It is proposed that the salt-tolerant variety 37 potentially mitigates intracellular Na^+^ toxicity via a more efficient ion transport system, such as Na^+^ efflux or compartmentalization mechanisms, possibly regulated by specific signaling pathways. Subsequent transcriptome analysis revealed enrichment of the “potassium ion homeostasis” pathway in the salt-tolerant variety 37 (**Supplementary Figure S5**), supporting the hypothesis that differential gene expression related to ion transport may underlie the divergent salt tolerance between the two varieties.

### 4.2 Potential regulatory role of seed endophytic communities in salt tolerance

Plant endophytic microorganisms play a crucial role in enhancing salt tolerance by modulating host nutrient uptake, hormone regulation, and stress signaling pathways (Gupta et al., 2021; Liu et al., 2022). Analysis of 16S rRNA sequencing data in this study revealed notable distinctions in the endophytic microbial communities between the salt-tolerant variety 37 and the salt-sensitive variety 44 under salt stress conditions. Specifically, the abundance of the dominant genus *Brevundimonas* in salt-tolerant variety 37 increased significantly from 30.00% to 45.27% under salt stress, and this increase was positively correlated with K^+^ content. In contrast, the abundance of the dominant genus *Rhodococcus* in salt-sensitive variety 44 increased to 64.96% under salt stress, exhibiting a positive correlation with Na^+^ content and the Na^+^/K^+^ ratio. These results highlight the association between specific microbial taxa and host plant salt tolerance. Previous studies have highlighted the capacity of *Brevundimonas* to enhance potassium absorption and mitigate salt stress through the secretion of indole-3-acetic acid (IAA) and 1-aminocyclopropane-1-carboxylic acid (ACC) deaminase (Kumar and Gera, 2014; Singh et al., 2016). The enrichment of *Brevundimonas* in the salt-tolerant variety 37 likely aids in maintaining ion equilibrium by facilitating potassium uptake, thereby ameliorating salt-induced stress. Conversely, the increased abundance of *Rhodococcus* in the salt-sensitive variety 44 may be linked to sodium accumulation, although the precise underlying mechanism warrants further investigation. Blasco et al. demonstrated the halotolerant nature of *Rhodococcus* sp. RB1, showing its ability to thrive in a medium with a high salt concentration of up to 0.9 mol/L NaCl or KCl. Upon the introduction of 0.5 mol/L NaCl, the bacterium exhibited a swift response by actively expelling K^+^ ions while simultaneously absorbing Na^+^ ions (Blasco et al., 2001). Furthermore, the salt-tolerant variety 37 and the salt-sensitive variety 44 exhibited 252 and 364 unique ASVs under salt stress, respectively-suggesting that salt stress may shape the host’s salt-tolerant phenotype by selecting specific microbial taxa.

### 4.3 Salt tolerance regulatory pathways revealed by transcriptome and WGCNA

Transcriptomic analysis revealed distinct enrichment pathways of DEGs under salt stress between salt-tolerant variety 37 and salt-sensitive variety 44. DEGs in the salt-tolerant variety 37 were predominantly enriched in stress defense pathways related to “ion balance” and “oxidative stress response”, whereas DEGs in the salt-sensitive variety 44 were primarily associated with substance metabolic processes such as “sucrose/starch catabolism” and “cell wall synthesis”. This suggests that salt-tolerant varieties may activate stress response pathways actively to combat salt stress, while salt-sensitive varieties rely more on fundamental metabolic adjustments (e.g., breaking down energy-storing compounds for energy supply) (Gogna and Bhatla, 2020; Jumpa et al., 2023). Feng et al. found that the salt-tolerant ryegrass variety ‘Abundant’ actively regulates stress responses such as ion uptake and transport to resist salt stress, while the salt-sensitive ryegrass variety ‘Angus’ mainly makes adjustments at the metabolite level (Feng et al., 2021). However, this passive response may be insufficient to mitigate stress damage, consistent with their observable phenotypic disparities. Weighted Gene Co-expression Network Analysis (WGCNA) categorized the DEGs into 5 modules, with the MEyellow module showing a significant positive correlation with Na^+^ content (r = 0.59) and predominantly up-regulated genes in the salt-tolerant variety 37. Within this module, the top 10 core genes included *WRKY* (*HORVU.MOREX.r3.3HG0268090*) and *RPK* genes (*HORVU.MOREX.r3.4HG0331910*). Zheng et al. identified 86 *WRKY* genes in the highland barley genome and experimentally validated their induction by salt stress, implicating their role in regulating highland barley’s response to salt stress (Zheng et al., 2021). Acting as a cell-membrane receptor protein kinase, *RPK* can detect external salt signals and trigger downstream pathways (e.g., MAPK and ABA pathways) (Eckardt, 2009; Loo et al., 2022). The high expression of this *RPK* gene in the salt-tolerant variety 37 suggests its potential as a pivotal node in salt signal transduction, enhancing salt tolerance by regulating processes like ion transport and reactive oxygen species scavenging. As a core component of eukaryotic chromatin, histone H2B regulates gene transcription through post-translational modifications (e.g., monoubiquitination), thereby participating in plant responses to salt stress. Zhou et al. confirmed that histone H2B monoubiquitination (*H2Bub1*) affects salt stress tolerance in *Arabidopsis thaliana* by regulating microtubule depolymerization-mutants defective in *H2Bub1* showed significantly increased salt sensitivity (Zhou et al., 2017). The DUF761 (Domain of Unknown Function 761) family consists of proteins with uncharacterized functions, but their role in plant stress responses can be inferred from expression patterns. Han et al. demonstrated that overexpression of a DUF gene in alfalfa (*Medicago sativa*) enhanced drought and salt tolerance (Han et al., 2025). Zhang et al. reported that overexpression of the *DUF761–1* gene in *Arabidopsis thaliana* altered leaf and root morphology, inhibited inflorescence stem elongation and thickening, and enhanced resistance to *Pseudomonas syringae* pv. tomato DC3000 (Zhang et al., 2019). GDSL esterases/lipases are a class of multifunctional hydrolases that participate in plant stress responses by catalyzing lipid metabolism, synthesizing signaling molecules, or producing antioxidants. Under salt stress, these enzymes can maintain membrane fluidity by regulating the lipid composition of cell membranes (e.g., increasing the proportion of unsaturated fatty acids) or activate stress defense pathways by generating lipid-derived signaling molecules (e.g., jasmonic acid precursors) (Jiang et al., 2012). Germin-like proteins (GLPs) are extracellular proteins with diverse enzymatic activities (e.g., oxidase, hydrolase) and are widely involved in plant abiotic stress responses. In potato (*Solanum tuberosum*), GLP family genes (e.g., *StGLP5*) are significantly up-regulated under salt stress, and their expression levels are positively correlated with antioxidant capacity-GLPs reduce salt-induced oxidative damage by catalyzing ROS scavenging (Zaynab et al., 2022). Late Embryogenesis Abundant (LEA) proteins are key protective proteins in plant stress responses, characterized by high hydrophilicity. They enhance salt tolerance through multiple mechanisms: 1) acting as molecular chaperones to maintain protein structural stability and prevent salt-induced protein denaturation; 2) binding water molecules or ions to maintain cellular osmotic balance; and 3) scavenging ROS to alleviate oxidative damage (Bhardwaj et al., 2013; Mohanty and Hembram, 2025). Proteins of the ALMT (Aluminium-Activated Malate Transporter) family are primarily responsible for mediating the transmembrane transport of organic acids such as malate. Traditional research on ALMT proteins has focused on their role in aluminum stress responses, but recent studies have confirmed their involvement in regulating salt stress. Under salt stress, ALMT proteins can secrete malate to chelate extracellular Na^+^-reducing Na^+^ influx into cells-and malate acts as an osmotic regulator to maintain cell turgor (Wu et al., 2025).

### 4.4 Characteristics and salt tolerance mechanisms of the RPK gene family in highland barley

In this study, 174 *HvRPK* genes were identified for the first time at the whole-genome level, distributed across 7 chromosomes, with a notable enrichment of 40 genes on chromosome 2H, indicating a clustering distribution. These genes exhibit physicochemical properties (e.g., transmembrane helices, signal peptides), domains (e.g., protein kinase domain, LRR repeats), and cis-elements responsive to ABA, drought, and salt stress-consistent with the functional characteristics of membrane receptors involved in signal transduction. For phylogenetic analysis, 145 *HvRPK* genes (after excluding those with incomplete motifs) were classified into 3 groups, with Group C being the largest (85 genes) and further subdivided into 3 subgroups, suggesting potential functional divergence associated with distinct stress response requirements. Collinearity analysis revealed high conservation of *HvRPK* genes between highland barley with and wheat/rice, with limited collinearity with *Arabidopsis*, indicating core functions retention, such as basic signal transduction, across angiosperm evolution, with specific expansion in gramineous plants possibly linked to adaptive responses to environmental stresses. Analysis of promoter cis-elements showed that 85.06% of *HvRPK* genes contain ABA-responsive elements and 74.71% contain stress-defense elements, implicating their involvement in salt stress signaling. ABA, a key signaling molecule in salt stress responses, may activate downstream salt-tolerance pathways by inducing *RPK* gene expression (Ye et al., 2022). Specifically, the expression levels of *RPK* genes, like *HORVU.MOREX.r3.4HG0331910*, was significantly higher in the salt-tolerant variety 37 compared to the salt-sensitive variety 44, with expression patterns negatively correlated with Na^+^ content, suggesting a role in maintaining ion balance by inhibiting Na^+^ accumulation or enhancing K^+^ uptake. This finding aligns with reports in *Arabidopsis* indicating that *RPK* gene triggers ion-homeostasis regulatory pathways by sensing salt signals (Shi et al., 2014). Nonetheless, further validation is needed to identify the specific downstream target genes of *RPK* in highland barley.

### 4.5 Putative mechanisms of salt-tolerant germination in highland barley

Salt stress is a major environmental constraint limiting crop seeds germination and seedlings establishment. This study focused on highland barley (*Hordeum vulgare* var. *coeleste Linnaeus*) and investigated the mechanisms of salt-tolerance germination, focusing on two key aspects: endophyte-mediated ion balance regulation and host gene signal transduction. 16S rRNA sequencing analysis revealed a significant increase in the relative abundance of the endophytic bacterium *Brevundimonas* in salt-tolerant variety 37 under salt stress (45.27%) compared to salt-sensitive variety 44 (4.05%). Subsequent analysis showed that intracellular Na^+^ content was lower in *Brevundimonas*-enriched variety 37 (4.24 g/kg) than that in variety 44 (4.89 g/kg). We hypothesize that *Brevundimonas* secretes metabolites (e.g., IAA, organic acids) to induce up-regulation of Na^+^/H^+^ antiporter expression in the host root cell membrane-facilitating intracellular Na^+^ efflux, mitigating salt-induced ion toxicity, and establishing a stable physiological foundation for seed germination. Transcriptomic analysis revealed a significant up-regulation of the membrane-localized *RPK* gene (*HORVU.MOREX.r3.4HG033191*) in salt-tolerant variety 37 compared to salt-sensitive variety 44 under salt stress. We propose that upon sensing increased extracellular salt concentrations, the *RPK* gene triggers its intracellular kinase domain, leading to the phosphorylation of crucial elements within the downstream MAPK pathway (e.g., MAPKKK, MAPKK). Subsequently, the activated MAPK translocates to the nucleus, where it phosphorylates transcription factors like WRKY and MYB, thereby modulating the expression of salt tolerance-related genes downstream. The salt-tolerant germination of highland barley seeds under salt stress is attributed to the synergistic interaction between *Brevundimonas* and the *RPK* gene: metabolites produced by *Brevundimonas* likely up-regulate *RPK* gene expression, thereby enhancing the activation efficiency of the signal transduction pathway. Concurrently, the *RPK* gene-induced MAPK pathway not only directly modulates salt tolerance gene expression but also potentially amplifies the regulatory effect of *Brevundimonas* on ion balance by increasing the expression of ion transporter genes. While direct verification of *Brevundimona*s function via pure culture inoculation experiments is pending, current findings preliminarily establish its pivotal role in seed germination under salt stress. Subsequent research can validate its direct regulatory influence through re-inoculation experiments in axenic systems.

## Data Availability

The datasets presented in this study can be found in online repositories. The names of the repository/repositories and accession number(s) can be found in the article/**Supplementary Material**.
