# Supplementary figures and images for: Integrative 16S rRNA and transcriptome analysis reveals the molecular mechanisms underlying salt- tolerant germination in highland barley (Hordeum vulgare var. coeleste Linnaeus) seeds
Source: Front Plant Sci. 2025 Nov 6;16:1691647. doi: 10.3389/fpls.2025.1691647 (PMC12629937; doi:10.3389/fpls.2025.1691647)

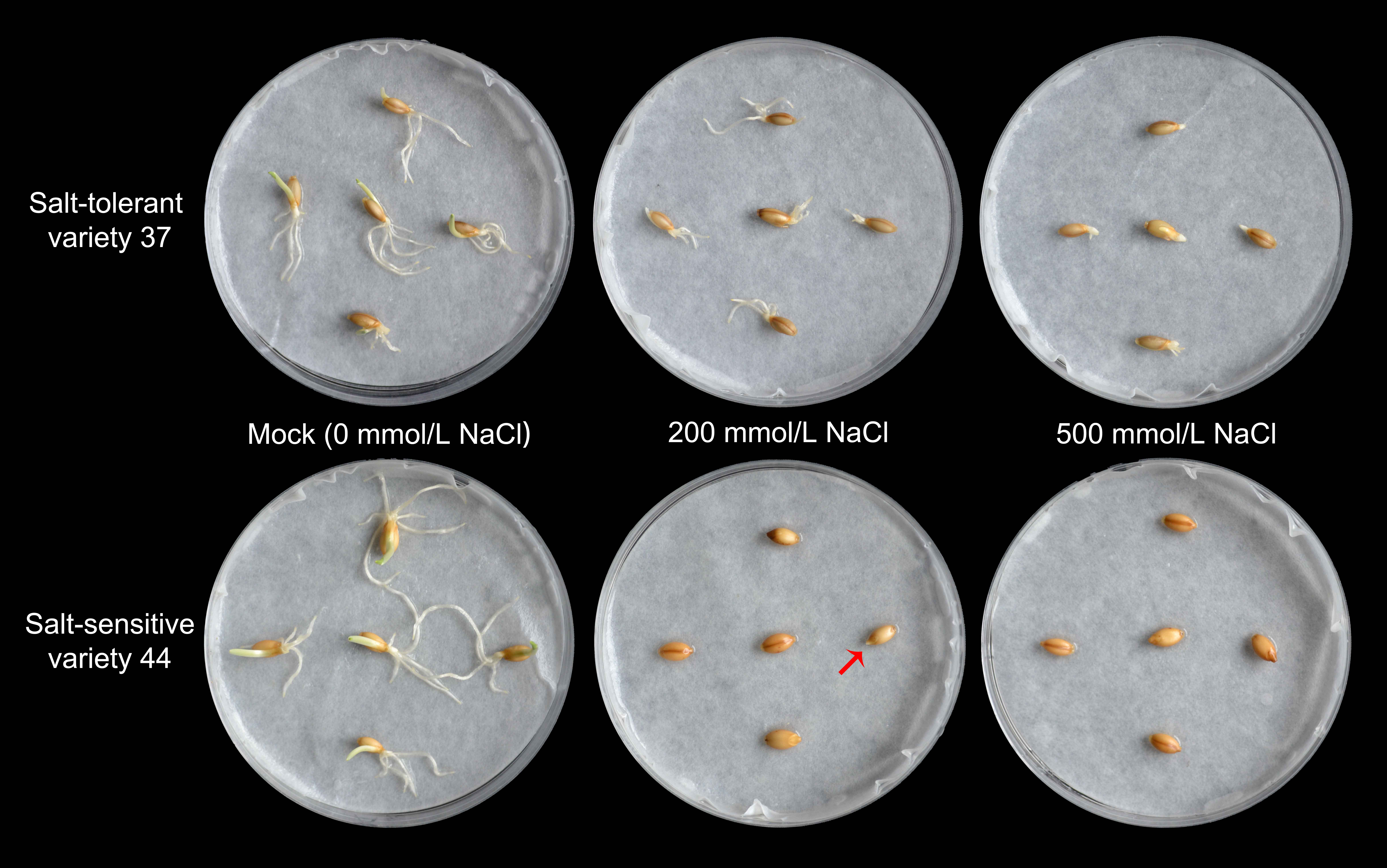

Supplement: Supplementary file 1 [file DataSheet1.zip › Supplementary figure 1-12/Fig-S1.jpg]
