# Supplementary material for: Integrative 16S rRNA and transcriptome analysis reveals the molecular mechanisms underlying salt- tolerant germination in highland barley (Hordeum vulgare var. coeleste Linnaeus) seeds
Source: Front Plant Sci. 2025 Nov 6;16:1691647. doi: 10.3389/fpls.2025.1691647 (PMC12629937; doi:10.3389/fpls.2025.1691647)
Supplement: Supplementary file 4 [file DataSheet4.docx]

Supplementary Material

# Supplementary Data

RNAseq (SRR33981615, SRR33981612, SRR33981614, SRR33981611, SRR33981613, SRR33981610, SRR33981609, SRR33981608, SRR33981606, SRR33981607, SRR33981605, SRR33981604, https://www.ncbi.nlm.nih.gov/Traces/study/?acc=SRP592153&o=acc_s%3Aa) and 16S rRNA sequencing (SRR33983123, SRR33983120, SRR33983117, SRR33983122, SRR33983119, SRR33983116, SRR33983124, SRR33983121, SRR33983118, SRR33983114, SRR33983113, SRR33983115, https://www.ncbi.nlm.nih.gov/Traces/study/?acc=SRP592187&o=acc_s%3Aa) data are available on the NCBI website.

# Supplementary Figures and Tables

## Supplementary Figures

**Supplementary Figure 1.** Images of salt-tolerant variety 37 and salt-sensitive variety 44 seeds under mock (0 mmol/L NaCl) and salt stress (200 mmol/L and 500 mmol/L NaCl).

**Supplementary Figure 2.** Relative abundance of different microbial groups in salt-tolerant variety 37 under salt stress.

**Supplementary Figure 3.** Relative abundance of different microbial groups in salt-sensitive variety 44 under salt stress.

**Supplementary Figure 4.** Correlation analysis on the germination rate, ion content (Na^+^, K^+^, Na^+^/K^+^ ratio) and abundance of five dominant genera in barley seeds.

**Supplementary Figure 5.** GO terms of DEGs in salt-tolerant variety 37.

**Supplementary Figure 6.** GO terms of DEGs in salt-sensitive variety 44.

**Supplementary Figure 7.** Screen of soft threshold.

**Supplementary Figure 8.** Check of soft threshold.

**Supplementary Figure 9.** Gene clustering tree of gene co-expression network.

**Supplementary Figure 10.** Heatmap of module-trait relationships (germination rate).

**Supplementary Figure 11.** The position of *HvRPK* gene family members on chromosomes.

**Supplementary Figure 12.** Distribution of CDSs and UTRs of *HvRPK* gene family members.

**Supplementary Figure 13.** Domain analysis of *HvRPK* gene family members.

**Supplementary Figure 14.** Conserved motifs analysis of *HvRPK* gene family members.

**Supplementary Figure 15.** Analysis of cis-elements in the promoter of *HvRPK* gene family members.

## Supplementary Tables

**Supplementary Table 1.** Germination data of 217 highland barley materials in different concentrations of NaCl solutions.

**Supplementary Table 2.** The Primers of qRT-PCR analysis.

**Supplementary Table 3.** Sequencing data volume and quality control of samples.

**Supplementary Table 4.** The alpha diversity of samples.

**Supplementary Table 5.** *HvRPK* gene family gene and protein properties.
